# Cognitive assessment of children with acute lymphoblastic leukemia: Preliminary findings

**DOI:** 10.4103/0971-5851.56330

**Published:** 2009

**Authors:** Anna Abraham, L. Appaji

**Affiliations:** 1 *Clinical Psychology Unit, Kidwai Memorial, Institute of Oncology (KMIO), Bangalore, Karnataka, India*; 2 *Department of Paediatric Oncology, Kidwai Memorial, Institute of Oncology (KMIO), Bangalore, Kanataka, India*

**Keywords:** *Acute lymphoblastic leukemia*, *children*, *cognitive functions*

## Abstract

**Aim:**

The objective of this study was to assess the cognitive functions of Indian children with acute lymphoblastic leukemia (ALL), periodically after initiation of treatment since prospective longitudinal research in this area on the Indian population has not been adequately documented. Unlike many western studies that have targeted survivors of ALL, we aimed to bring out the cognitive outcome after initiation of treatment.

**Materials and Methods:**

The cognitive functions of 19 patients diagnosed to have ALL were assessed using standardized tests after induction chemotherapy, and periodically thereafter following the second course of treatment comprising central nervous system-directed radiotherapy, and chemotherapy using intrathecal methotrexate.

**Results:**

The study found a statistically significant decline in the intelligence quotient and a deficit in the cognitive function of analytical reasoning.

**Conclusion:**

This preliminary study supports findings of an earlier Indian study and many studies conducted in the west. Since the life expectancy of these children has increased and most of them have long-term survival, and even cure, we suggest that identifying and managing children with cognitive difficulties are important in the rehabilitation of these children.

## INTRODUCTION

Until four decades ago, children diagnosed to have acute lymphoblastic leukemia (ALL) had a life expectancy of less than 1 year. Death often resulted not from the original hematological disease but from its progression within the central nervous system (CNS).[1] Administration of CNS-directed radiotherapy (RT) and intrathecal methotrexate (ITMTX) has dramatically increased the life expectancy of these children.[2] Now, about 70% can expect long-term survival and even cure.[3] Although such treatment programs have clearly resulted in prolonged disease-free survival for children with ALL, CNS sequelae have been reported, particularly where treatment regimens included cranial irradiation.[4] This evidence is clear for the cognitive outcome in survivors of ALL in western countries. Studies on the cognitive outcome after initiation of treatment are relatively scarce.

During long-term follow-up of children treated for ALL that had CNS-directed RT [18 Grey (Gy) in 10 fractions] and chemotherapy using ITMTX in our institution, there were reports from parents that children were unable to attend to simple tasks or their eye-hand coordination was poor or their memory was unsatisfactory or their arithmetic ability was inadequate. These reports were substantiated by the school teachers′ reports of these children. This observation was further supported by cognitive deficits found on objective psychometric tests. Hence, we attempted a systematic prospective longitudinal study, since research in this area on the Indian population has not been adequately documented. The objective of the present study was to assess the cognitive functions of Indian children with ALL, periodically after initiation of treatment. The present study, in contrast to many western studies that have targeted survivors of ALL, has attempted to bring out the cognitive outcome after initiation of treatment.

## MATERIALS AND METHODS

### Patient recruitment

A total of 371 ALL patients (children) were sent to the clinical psychologist over a period of 6½ years.

A sample of 77 patients from this pool of 371 children diagnosed to have ALL from the Paediatric Oncology Department of our institution was included in the study. Inclusion criteria were patients 6-12 years old and knowing the local language, i.e., Kannada. Exclusion criteria were patients with history of CNS disease; sensory motor deficits; and a concurrent neurological or psychiatric disorder and/or mental retardation (MR). The remaining 294 patients could not be included in the study due to not fulfilling selection criteria, not reporting after first contact, not keeping appointments or not being cooperative for assessments.

The 77 patients were fairly homogeneous in terms of the socioeconomic status, i.e., they mostly belonged to the lower socioeconomic status group. Financial aid was mobilized through several resources available.

In the Indian population, family ties are apparently strong and it is the cultural practice to bond closely, especially in a crisis such as caring for a sick child. Parents and other significant persons were counseled regarding handling of stress and the need to provide emotional support to the sick child. All efforts were made to create a homely atmosphere for the patient during hospitalization. Return to school as soon as possible was advised so that the children would be able to get back into a normal routine as before. Thus, these patients were given apparently adequate care before, during and after treatment. With regard to the above, it is apparent that the sample of 77 patients was also homogeneous in terms of family/social relationships and emotional bonding.

### Design of the study

A single-group outcome study with multiple assessments was used where each patient was his or her own control. All 77 patients were treated with a multicentric protocol (MCP 841) of the National Cancer Institute.

### Experimental procedure

After diagnosis and the first course of treatment, i.e., induction chemotherapy, and before the commencement of the second course of treatment (CNS-directed RT with ITMTX), patients without history of CNS disease, sensory motor deficits and a concurrent neurological disorder were sent to the clinical psychologist. Patients fulfilling the remaining selection criteria were then included in the study. The rationale, nature, aims and implications of the study were then explained to the parent(s), and their consent to participate in the study was obtained.

Subsequently, psychological tests appropriate for this 6-12 year age group were chosen: The Bender gestalt test (BGT) for young children;[5] colored progressive matrices (CPM);[6] and the Binet Kamat test (BKT) of intelligence. [7] The BGT is a nonverbal test of visuo-spatial perception and visuo-motor coordination and integration. It consists of simple geometrical figures which the subject has to copy as they are presented one by one. The test is scored on the basis of certain errors made. The total number of errors made provides an age equivalent, which is a measure of the cognitive function. The CPM is a nonverbal, culture-free test. It is designed to assess a child′s ability to form comparisons and reason by analogy. It comprises three sets of 12 problems each, represented as patterns with a missing piece, where the subject has to find the missing piece from the alternatives given. It provides a percentile score on analytical reasoning, which is also a measure of the child′s intelligence level. The BKT measures general intelligence and has been standardized on Indian children, a Kannada version being available. It provides a global IQ (verbal and nonverbal) of the subject. Pattern analysis of the test items provides estimates of specific cognitive functions. These tests were chosen since the BGT and CPM are nonverbal/culture-free tests for easy use with Indian patients; and the BKT has been standardized on Indian children, with a Kannada version for use with the Kannada-knowing patients. Moreover, data was collected during a period when there was no comprehensive test or battery of tests standardized on Indian children in the Kannada language available. The psychological tests were administered by the clinical psychologist; and as far as possible, the patients were not tested on days when other medical procedures were scheduled. The sessions were also spaced out to overcome the effect of fatigue. Testing was usually completed in 2-3 days, 1-2 sessions per day, each session lasting for 30-40 minutes. This assessment constituted the first assessment. It was completed before the commencement of the second course of treatment.

After completion of the second course of treatment, i.e., CNS-directed RT (18 Gy in 10 fractions) and ITMTX, patients were assessed periodically, resulting in multiple assessments. There were three further assessments that were identical to the initial psychological assessment, with an interval between assessments of at least 6 months to overcome practice effect. During these subsequent assessments, the patients were either on maintenance treatment or follow-up in an outpatient clinic. The assessments, however, did not interfere with the routine treatment protocol for ALL while on treatment and with investigative medical procedures while on follow-up. Patients were sent to the clinical psychologist whenever they came to the outpatient clinic. Patients did not have CNS disease or systemic relapse of ALL during these assessments.

### Statistical analysis of data

Only the data of patients that were repeatedly assessed were considered for statistical analysis. The nonparametric test, viz., Wilcoxon′s signed-rank test, for paired sample differences[8] and the parametric test, viz., Student *t* test, for comparing means of two correlated samples[8] were used for statistical analysis. A *P* value less than 0.05 was considered statistically significant.

## RESULTS

Seventy-seven patients that were included in the study completed the first assessment. However, at the second assessment, 19 patients completed the BGT and CPM; and of the 19, 11 patients completed the BKT. Seven and 3 patients out of the 19 completed all the tests at the third and fourth assessments, respectively. The 19 patients that were available for the second assessment were apparently similar to the remaining 58 patients in the various socio-demographic variables.

The marked decline in the number of patients over the duration of the study was due to several psycho-social factors such as time constraints, long distance of travel, family/social responsibilities, etc.

Thus, 19 patients from the initial pool of 77 patients that could be repeatedly assessed were considered for statistical analysis. Of the 19 patients studied, 14 were males and 5 were females. The mean age of the group was 9.46 ± 1.56 years. The second assessment was carried out about 10 months (mean, 10.47  ±  5.21) after the first assessment. The range was 6-22 months. The third assessment was done about 12 months (mean, 12.14 ± 6.62) after the second assessment and 23 months (mean, 23.21 ± 11.92) after the first assessment. The ranges were 6-22 and 12-44 months, respectively.

Table 1 shows the level of significance of the *t* values for paired sample differences in the variables as measured by the BGT and CPM between the first and second assessments. As mentioned earlier, there were 19 patients that completed the BGT and CPM at the second assessment. As given in the table, there was no significant change in the age equivalent scores on the BGT, although an increment in the scores was evident at the second assessment as compared to the first. However, there was a significant decrease in the CPM values at the 0.01 level at the second assessment as compared to the first. The comparison of mean values of the IQ as measured by the BKT between the first and second assessments is shown in [Table 2]. As mentioned earlier, there were 11 patients that completed the BKT at the second assessment. As indicated in the table, there was a significant decrease in the BKT values at the second assessment as compared to the first. Table 3 shows the level of significance of the *t* values for paired sample differences in the variables as measured by the BGT and CPM between the second and third assessments. As mentioned earlier, 7 patients completed both the tests at the third assessment. As indicated in Table 3, there was no significant change in the BGT scores, although an increment in the scores was obtained at the third assessment as compared to the second. However, as in Table 1, the CPM percentile scores declined significantly at the third assessment as compared to the second [Table 3]. The comparison of mean values of IQ as measured by the BKT between the second and third assessments is shown in Table 4. As mentioned earlier, there were 7 patients that completed this test at the third assessment. As indicated in the table, there was a significant decrease in the BKT values at the third asvsessment as compared to the second.

**Table 1:** Level of significance of the *t* values for paired sample differences in the variables as measured by the Bender Gestalt test and colored progressive matrices between the first and second assessments

Psychological	Variable	Level of significance
test		(2 tailed)
BGT	Age	*P* > 0.05 NS
n = 19	equivalent	
m = 14	(in months)	
f = 5		
CPM	Percentile	*P* < 0.01 *
n = 19	score	
m = 14		
f = 5		

BGT = Bender Gestalt test, CPM = colored progressive matrices, n = number of patients, m = males, f = females, NS = not significant,

* = significant

**Table 2:** Comparison of mean values of intelligence quotient measured by the Binet Kamat test between the first and second assessments

Psychological	Variable	Mean ± SEM		*P* value
test		Mean_ 1_ M_1_	Mean_ 2_ M_2_	
BKT	IQ	92.68 ± 4.80	85.72 ± 3.58	*P* < 0.05 *
n = 11				
m = 7				
f = 4				

BKT = Binet Kamat test, SEM = standard error of mean, n = number of patients, m = males, f = females, IQ = intelligence quotient

**Table 3:** Level of significance of the *t* values for paired sample differences in the variables as measured by the Bender Gestalt test and colored progressive matrices between the first and second assessments

Psychological test	Variable	Level of significance (2 tailed)
BGT	Age equivalent	*P* > 0.05 NS
	(in months)	
n = 7		
m = 4		
f = 3		
CPM	Percentile score	*P* < 0.05 *
n = 7		
m = 4		
f = 3		

BGT = Bender gestalt test, CPM = colored progressive matrices, n = number of patients, m = males, f = females, NS = not significant,

* = significant

At the fourth assessment, the number of patients reduced to 3. Since there were only 3 patients at the fourth assessment, the findings on this assessment could not be statistically analyzed.

## DISCUSSION

Unlike many western studies that have targeted survivors of ALL, the present study aimed to bring out the cognitive outcome after initiation of treatment. As pointed out earlier, psychological assessment of ALL children in a prospective longitudinal study has many practical problems. Since they came from faraway rural areas, the follow-up was difficult; and in view of their illness, it was sometimes difficult to engage them for long periods of time due to easy fatigability. However, the present study was an attempt to systematically examine the cognitive outcome of these children since prospective longitudinal research in this area on the Indian population has not been adequately documented.

In the present study, on the BGT, comparing the first and second; and second and third assessments [Tables 1 and 3], there was no evidence to indicate that the BGT age equivalent scores significantly changed, although an increment in the scores was seen at the second and third assessments as compared to the first and second, respectively, indicating an improvement in the cognitive function. Previous studies done in western countries have shown that CNS changes take place several months after cranial irradiation and that deficits are often in the areas of fine motor coordination and visuo-spatial ability. In the present study, such deficits were not seen. Likewise, the Indian study by Jain *et al*.,[9] did not find a significant change on the BGT scores. This discrepancy could have been due to the different psychometric measures used. Moreover, the BGT is a developmental test and its function is determined by the biological principles of sensory motor action, and varies depending on the growth pattern and maturation level of an individual and his or her pathological state, functional or organic.[5] This could explain the increment in the BGT outcome in our study.

On the CPM, comparing the first and second; and second and third assessments [Tables 1 and 3], there was evidence to indicate that the percentile scores significantly declined at the second and third assessments as compared to the first and second, respectively. According to the literature, the function of analytical reasoning measured by the CPM appears to be one of the earliest to decline due to organic involvement.[6] 

**Table 4:** Comparison of mean values of intelligence quotient measured by the Binet Kamat test between the first and second assessments

Psychological	Variable	Mean ± SEM		*P* value
test		Mean_ 2_ M_2_	Mean_ 3_ M_3_	
n = 7, m = 4, f = 3	IQ	87.34 ± 5.63	81.94 ± 5.04	*P* < 0.05 *

BKT = Binet Kamat test, SEM = standard error of mean, n = number of patients, m = males, f = females, IQ = intelligence quotient

On the BKT, comparing the first and second assessments, there was evidence suggesting that the IQs of subjects had significantly decreased after the second course of treatment [Table 2]. The IQ decline was from an average to a high normal level.[7] To find out whether there would be a further decline in the IQs, the second and third assessments were compared [Table 4]. A significant decline at the 0.05 level was obtained. The IQ decline was from a high normal level to a low normal level.[7] The overall decline in IQ was from an average to a low normal level.[7] 

These preliminary findings support earlier work done in India by the All India Institute of Medical Sciences (AIIMS)[9] and in the west, where declines in intelligence levels[10,11] and IQs[12–14] have been reported after cranial irradiation. In most of the western studies, since survivors of ALL have been the population targeted for research, one could argue whether variables like decreased family/ social relationships and emotional bonding could have confounded the findings of the present study, since the patients were assessed after initiation of treatment, during hospitalization and at frequent visits to the hospital thereafter. However, considering the cultural practice of family bonding, especially in crisis situations, unique to the Indian population and advice regarding return to school as soon as possible, these variables should not have confounded the findings. Declines in the intelligence level and IQ in the present study were reported, on an average, after 10 months of cranial irradiation (18 Gy) (refer to text). This supports previous work done in the west.[15] Some studies, however, suggest that a longer time period is needed, maybe even years, for declines to be apparent,[16,17] especially with lower radiation doses.

It was apparent from a qualitative pattern analysis of the cognitive functions on the BKT across the first to third assessments, that the cognitive functions maximally affected were mainly nonverbal functions, those of conceptual thinking and nonverbal reasoning; while those of numerical reasoning and visuo-motor coordination were affected to a lesser extent. The CPM, as mentioned earlier, showed evidence for a statistically significant decline in analytical reasoning [Tables 1 and 3], one of the functions earliest to decline with organic involvement. [6] Western research that has identified deficits after cranial irradiation indicates that problems are often in the areas of nonverbal activities, particularly fine motor coordination, visuo-spatial ability and somatosensory functioning.[16,18–20] This discrepancy in the types of deficits seen could be due to these previous studies differing considerably in methodology, even in the choice of psychometric measures. Deficits in information-processing abilities, like attention, concentration, new learning and memory.[12,14,17,21–23,24–26] have also been reported, especially with higher doses of cranial irradiation, i.e., of 24 Gy.[12,21–23,25] Even in the Indian study by Jain *et al*.,[9] deficits in attention, concentration and memory were reported, in which a higher dose of cranial irradiation, i.e., of 20 Gy, was used. These were not apparent in the present study, which used a lower dose.

Previous western studies[4] have shown that the identified deficits are not usually severe, particularly with regard to intellectual abilities, where many children treated with cranial irradiation function within the average range on psychometric tests. Our findings on tests other than the Wechsler Intelligence Scale for Children – Revised (WISC-R)[27] suggest that patients function within the low normal range.

Since the life expectancy of these children has increased and most children have long-term survival and even cure, we suggest that identifying children with cognitive difficulties and parental counseling[25] could be important in the rehabilitation of these children, so that they may benefit from early educational intervention[25] directed towards using cognitive strengths to overcome difficulties. An individualized program[25] which uses a multisensory method of teaching[21] may compensate for weaker abilities. In exceptional cases, admission into special schools for slow learners[3,28] is another option, where remedial training in scholastic skills can be tried. [3,26] If possible, readmission into a normal school with language exemption, which is possible in some states of the country, can be considered; or else a school leaving certificate can be obtained from the National Open Schools.[29] 

The present study is undoubtedly preliminary in nature, considering that the findings are based on a small sample of patients. Moreover, the tests used for the assessment of cognitive functions are rarely used currently, especially for research. Hence, the use of newer and more comprehensive batteries of tests, with vernacular translations, standardized on Indian children, on a larger sample; and comparing outcomes with other socioeconomic groups are needed. Unlike the previous Indian study,[9] which was a cross-sectional one, the present study is a prospective longitudinal one and highlights the need for identifying and managing children with cognitive difficulties for long-term rehabilitation. Interestingly, the Indian study by AIIMS[9], while making suggestions for future research, commented that psychometric evaluation at regular intervals is essential for long-term rehabilitation. The present study has attempted to fulfill this suggestion. However, for conclusive evidence, it is important to study the nature of these cognitive difficulties and the factors that contribute to a worse outcome. Long-term follow-up of these children will reveal whether these difficulties remain constant, intensify or resolve over time.[14] Studies from a purely neuropsychological perspective, with the inclusion of cognitive retraining as a part of the rehabilitation of these children, may also be tried.

In conclusion, given that the sample was apparently homogeneous in terms of the socioeconomic status, family/social relationships, and emotional bonding, the present study, though preliminary in nature, gives evidence for a significant IQ decline and a deficit in the cognitive function of analytical reasoning in children with ALL that have undergone CNS-directed RT and ITMTX. Further research in this area is warranted.
